# Quinine and Insomnia

**Published:** 1913-09-27

**Authors:** 


*To the Editor of* The Hospital.

### Quinine and Insomnia.

*To the Editor of* The Hospital.

Sir,—I read in The Hospital for September 13 an article on insomnia. In the whole article there is no mention of quinine as a help to sleep. Surely it ought to be tried always before resorting to any of the drugs suggested, which should be avoided if possible. Why is it apparently so seldom prescribed by doctors for sleeplessness when it has such a splendid effect in so many cases? A friend of mine was recently in a nursing home, and was sleeping badly after an operation. She asked for quinine, as she knew it always made her sleep better. Neither doctor nor nurses had ever apparently given it before to induce sleep, but it had a splendid effect. Seeing this, the patient's night nurse, who was sleeping very badly, also took some. She immediately had a very good night. The day nurse then tried it, but said never again would she take it, as she was much too sleepy next morning. One or two grains at bedtime should be taken. Many people who think they cannot take it without having a headache will find they can at night.—Yours faithfully,

F. A. M.

[Quinine does not seem to be generally known to or used by the medical profession as a hypnotic. We doubt whether it can have this property, except in occasional or unusual cases, but we are quite prepared to keep an open mind on the matter, in view of "F. A. M.'s" experience. Perhaps some of our medical readers can throw light on this particular remedy for insomnia.—Ed. The Hospital.]

J. D. G.—Your letter *re* Hospital Telephones is being attended to, and an answer will be sent very shortly.

